# Contribution of Angiogenesis to Inflammation and Cancer

**DOI:** 10.3389/fonc.2019.01399

**Published:** 2019-12-12

**Authors:** Dolores Aguilar-Cazares, Rodolfo Chavez-Dominguez, Angeles Carlos-Reyes, César Lopez-Camarillo, Olga N. Hernadez de la Cruz, Jose S. Lopez-Gonzalez

**Affiliations:** ^1^Departamento de Enfermedades Cronico-Degenerativas, Instituto Nacional de Enfermedades Respiratorias “Ismael Cosio Villegas”, Mexico City, Mexico; ^2^Posgrado en Ciencias Biologicas, Universidad Nacional Autonoma de Mexico, Mexico City, Mexico; ^3^Posgrado en Ciencias Genomicas, Universidad Autonoma de la Ciudad de México, Mexico City, Mexico

**Keywords:** inflammation, angiogenesis, carcinogenesis, cancer, vascular hyperpermeability, vasculogenic mimicry, metastasis

## Abstract

During carcinogenesis, advanced tumors are surrounded by both stromal and immune cells, which support tumor development. In addition, inflammation and angiogenesis are processes that play important roles in the development of cancer, from the initiation of carcinogenesis, tumor *in situ* and advanced stages of cancer. During acute inflammation, vascular hyperpermeability allows inflammatory mediators and immune response cells, including leukocytes and monocytes/macrophages, to infiltrate the site of damage. As a factor that regulates vascular permeability, vascular endothelial growth factor (VEGF) also plays a vital role as a multifunctional molecule and growth factor. Furthermore, stromal and immune cells secrete soluble factors that activate endothelial cells and favor their transmigration to eliminate the aggressive agent. In this review, we present a comprehensive view of both the relationship between chronic inflammation and angiogenesis during carcinogenesis and the participation of endothelial cells in the inflammatory process. In addition, the regulatory mechanisms that contribute to the endothelium returning to its basal permeability state after acute inflammation are discussed. Moreover, the manner in which immune cells participate in pathological angiogenesis release pro-angiogenic factors that contribute to early tumor vascularization, even before the angiogenic switch occurs, is also examined. Also, we discuss the role of hypoxia as a mechanism that drives the acquisition of tumor hallmarks that make certain cancers more aggressive. Finally, some combinations of therapies that inhibit the angiogenesis process and that may be a successful strategy for cancer patients are indicated.

## Introduction

According to Hanahan and Weinberg, cancer cells demonstrate 10 common properties, including the ability to evade growth suppressors, and avoid cell death, sustained cellular proliferation, replicative immortality, genomic instability, energetic cellular deregulation, the ability to suppress immune destruction, and induce angiogenesis, the ability to invade surrounding tissues and promote metastasis, and the ability to promote tumor-related inflammation (1). Tumors are comprised of both abnormal and normal cells, and this heterogeneous composition serves to maintain many complex, dynamic, and shifting interactions among the tumor, immune, and stroma cells in the microenvironment (2). The association between inflammation and cancer is widely recognized. In 1863, Rudolf Virchow (3, 4) reported that some tumors were infiltrated by inflammatory cells, leading to the hypothesis that inflammation is associated with cancer. In 2012, the International Agency for Research on Cancer established that infection with some pathogens, including *Helicobacter pylori*, human papillomavirus (VPH) variant 16, and *Schistosoma haematobium*, is associated with cancer. This and other observations support the notion that persistent infection and inflammation are concomitant with the process of carcinogenesis. In addition, chronic sterile inflammation induced by some non-infectious agents, such as asbestos, UV light, and silica crystals, may leads to cancer development (5–8).

Tumors have a high metabolic rate and require a constant supply of nutrients, along with the exclusion of waste material. These processes are successfully achieved through the induction of angiogenesis. To preserve physiological homeostasis, angiogenesis is rigorously linked with the inflammatory processes. However, in deregulated inflammatory processes that lead to chronic inflammation, pathological angiogenesis can be initiated (9). An intimate connection between immune cells and the endothelium occurs during inflammation. In addition, several studies have indicated that immune/endothelium cell interactions are maintained and encourage tumor development.

In this review article, we focus on mechanisms during acute inflammation that lead to vascular hyperpermeability. In addition, the development of pathological angiogenesis during chronic inflammation is discussed, highlighting the preservation of this process during carcinogenesis. Furthermore, how vascular hyperpermeability, angiogenesis, and inflammation work together in the development of cancer is examined. Therapeutic advances for the normalization of tumor vasculature are indicated. Finally, our particular vision in terms of the roles that angiogenesis and inflammation play in tumor development is presented.

## Acute Inflammation/Vascular Hyperpermeability

Inflammation is defined as the physiological response to infectious or non-infectious agents. The process of inflammation is activated in order to remove both damaged tissue cells and the source of injury (10). The overall goal of the inflammatory process is the reparation of damaged tissue in order to restore the typical tissue architecture, thus maintaining cellular/tissue homeostasis. During the inflammatory process, cells damaged by infectious agents, or cellular stress, release endogenous molecules known as alarmins or danger-associated molecular patterns (DAMPs) that translocate to the cell membrane. These DAMPs are then sensed by a wide variety of cells that express distinct pattern recognition receptors (PRRs), including toll-like receptors (TLRs) and nucleotide-binding oligomerization domain (NOD)-like-receptors (NLR) (11–15). In particular, leukocytes (M1 macrophages, monocytes, neutrophils, mast cells, eosinophils, and other cells) link DAMPs to induce the activation of the inflammasome and the NF-κB signaling pathway. Subsequently, these cell types release several pro-inflammatory cytokines, such as VEGF, IL-1α, IL-1β, and TNF-α, along with the chemokines IL-8, MIP-1α, and RANTES (16). Other inflammatory mediators, including bradykinin, histamine, thrombin, and fibrinogen, and endotoxins such as lipopolysaccaride (LPS) are also released. The target cells for these cytokines and chemokines, particularly those of VEGF/VEGFR, are endothelial cells, which then induce vasodilatation (edema) and increase vascular permeability (17, 18). In addition, the expression of several adhesion molecules, such as E-selectin, P-selectin, ICAM-1, ICAM-2, and VCAM-1, is initiated (19, 20). The activation of the endothelium as mediated by these factors is important for the passage (transmigration) of inflammatory cells from the blood to the site of damage (21). Studies have indicated that the increased permeability that occurs during the inflammation process is localized to the microvasculature, primarily in the post-capillary vein (22, 23).

### Vascular Hyperpermeability and the Role of VEGF

The endothelial barrier consists of the joining of endothelial cells by diverse lateral cell-cell junctions. These tight-junctions (TJs) involve specific molecules, namely, claudins and occludins, which form a zipper-like structure between cells that controls the paracellular passage of ions and solutes. TJs are found primarily in the blood-brain barrier (22, 24).

Adherens junctions (AJs) are another important union and are formed by cadherins and catenins molecules. AJs serve to maintain the cell-cell adhesive contact. The vascular/endothelial (VE) cadherin mediates homotypic adhesion with the adjacent cell in a calcium-dependent manner. The intracellular domain of the VE-cadherin is anchored to the cytoskeleton by means of various catenins (α, β, γ, and p120 catenins) that comprise the AJ (25, 26). In addition, intracellular catenins also transmit signals for cell-cell communication (26).

Endothelial cells are also tethered to the extracellular matrix (ECM) by focal adhesions mediated by a family of actin-like proteins, including focal adhesion kinase, talin, and paxillin (22, 25).

The hyperpermeability of the endothelium is mediated by cytokines and chemokines during the inflammatory process and is carried out by two transport mechanisms that facilitate the arrival of immune cells to the damaged area (27, 28). In this process, caveolin-dependent vesicles or vacuoles (vesiculo-vacuolar organelles or VVOs) form transendothelial channels in specialized regions of the plasma membrane (27–29). Through the sequential fusion of the VVOs, transcellular transport is allowed and used to deliver the contents of the VVOs to the extravascular space (25, 27, 28). This transport mechanism serves to carry proteins of 50–100 nm from the luminal area to the abluminal area of the endothelium (27). While the precise activation mechanism is not fully known, it has been reported that exposure of the endothelium to various factors, such as histamine and VEGF-A, results in the activation of VVO transport (25). Ultrastructural studies have suggested that VVOs form grape-like structures with interconnecting vesicles and vacuoles throughout cells (28, 29). In addition, it has been suggested that G proteins and members of the Src tyrosine kinase family are important for the signaling cascade involved in this transport mechanism (24, 25).

Another mechanism of transport involves a paracellular process (22, 26, 28). During this type of event, cell-cell endothelial junctions are temporarily inhibited, with several inflammatory mediators released into the circulation, including histamine, thrombin, VEGF, and pro-inflammatory cytokines (25, 26). Various signaling pathways, including those involving Rho GTPases, MAP kinases, and protein kinases, are then activated by these factors, leading to the interruption of cell-cell joints and the migration of phagocytic and other blood cells (25).

Although transendothelial transport occurs during inflammation in order to increase vascular permeability, paracellular transport is believed to be primarily involved in cell migration (22, 26).

Recent studies have indicated the importance of mural cells, including pericytes, smooth muscle cells, and macrophages, in the regulation of permeability (30–33). An excellent review of these aspects has been published by Goddard and Iruela-Arispe (34).

VEGF is the main soluble factor that modifies the endothelial barrier (35–37). This factor is secreted by neutrophils, platelets, macrophages, activated-T cells, dendritic cells, pericytes, and the endothelial cells themselves (38). VEGF was isolated in 1989 by Ferrera from the Genentech group (39). Several homodimeric glycoproteins comprise the VEGF family. In mammals, five members of the VEGF family have been identified, namely VEGF-A, VEGF-B, VEGF-C, VEGF-D, and placenta growth factor (PLGF) (36, 37, 40). As the prototypical VEGF, VEGF-A is considered the most potent stimulator of vasculogenesis and angiogenesis (38). In addition to increased vascular permeability, vasodilatation, and the recruitment of inflammatory cells, VEGF triggers the inhibition of apoptosis and increases cellular proliferation (38).

The biological activity of VEGF is mediated by the high affinity tyrosine kinase receptors VEGFR-1, VEGFR-2, and VEGFR-3. VEGFR-2 is expressed primarily in endothelial cells and its interaction with VEGF-A triggers increased vascular permeability. VEGFR-2 dimerization induces the autophosphorylation of tyrosine residues and the activation of specific signaling pathways, including the PI3K and p38 MAPK pathways (36, 37, 40). In addition, conformational changes induced by receptor dimerization lead to an increase in intracellular Ca^2+^, the activation of PLCγ and endothelial nitric oxide synthase (eNOS), with the latter resulting in increased production of nitric oxide (NO) (41, 42). In addition, Src kinase activation induces the phosphorylation of VE-cadherin and various catenins, preventing them from anchoring to the cytoskeleton (22, 25, 26).

Increased vascular permeability allows for platelets and immune cells such as neutrophils and monocytes/macrophages to reach the site of tissue damage (17). At the site of damage, platelets then participate in the coagulation process in order to prevent blood loss from damaged vessels (17). Subsequently, neutrophils arrive at the site of damage to eliminate the pathogen by means of reactive oxygen species (ROS) (43). Finally, the monocytes/macrophages arrive to phagocytose dead cells, cell debris, and various compounds of the ECM, including fibrin. In addition, neutrophils are removed by efferocytosis (44, 45). The resolution of the associated tissue damage and the return to a normal tissue structure with proper tissue-specific funcions are the goals of the vascular hyperpermeability associated with inflammation (22).

### Resolution of Vascular Hyperpermeability

The resolution of inflammation is a highly orchestrated process involving numerous biochemical processes. In order for this resolution to be successful, inflammatory mediators must act on specific targets to initiate a series of events resulting in homeostasis (46, 47). In particular, the actions that must be accomplished are as follows: (i) turning off the recruitment of neutrophils/lymphocytes, (ii) normalization of the cytokine gradient and the apoptosis of neutrophils, (iii) activation of apoptosis signals for leukocytes and the silencing of pro-inflammatory signaling pathways, (iv) efferocytosis by macrophages and the reprogramming of macrophages from classically activated (M1) to alternatively activated (M2), (v) incorporation of myeloid cells into the local population or recirculation by blood or lymphatic routes, and (vi) tissue repair/return to homeostasis and basal permeability (46, 47). The particular molecules responsible for carrying out the above events include the cytokines produced by M2 macrophages and specialized lipids such as lipoxins, resolvins, protectins, and maresins (48). Proteins such as annexin-A1, adrenocorticotropic hormone, galectin-1, and adenosine are also involved (46). These molecules are synthesized by various cell types, including neutrophils, macrophages, and endothelial cells.

Although the mechanisms by which the hyperpermeability of the endothelium returns to the basal state have yet to be completely described, oxidized phospholipids are known to act as protectors of the endothelial barrier (49). At low concentrations, the oxidized *1-palmitoyl-2-arachidonic-sn-glycerol-3-phosphorylcholine* (PAPC) (OxPAPC) inhibits TNF-α production in phagocytes by blocking the NF-κB pathway (49). In addition, OxPAPC is involved in the restoration of vascular permeability through the activation of the GTPases Cdc42 and Rac. This results in increased cortical actin, the stabilization of cell-cell junctions, and the inhibition of paracellular gap formation. Cdc42 and Rac also activate the Ras-associated protein-1 (Rap1) signaling pathway. Rap1 is an important regulator of various cell functions, including cellular polarization, and leads to increased VE-cadherin and β-catenin, as well as ZO-1 and ocluddin. Furthermore, OxPAPC interacts with the 78 kDa glucose-regulated protein GRP78, which is a multifunctional protein found in the endoplasmic reticulum and plasma membrane. This interaction then provides stability to the union of AJs with TJs (49–51).

## Angiogenesis in Chronic Inflammation

The persistence of the harmful agent that induced the inflammation leads to the upregulation of the inflammatory response. As already mentioned, vascular hyperpermeability promotes the presence of inflammatory cells such as monocytes and macrophages. These cells release pro-inflammatory cytokines, including TNF-α, IL-1β, and IL-6 that increase the expression of adhesion molecules and chemokines for further recruitment of T-lymphocytes (52). In these immune cells, activation of signaling pathways such as, NF-κB, MAPK, and JAK-STAT increase cytokines production. The arrival of more immune cells exacerbates the inflammatory response inducing a chronic inflammation. In response to these factors, the endothelial cells promote angiogenesis. The endothelial cells proliferate and migrate to form new capillaries contributing to restoring nutrient levels and facilitating immune cell migration (53).

In this shifting microenvironment, the immune cells gradually modify their cytokine profile sustaining the inflammatory network. In particular, the presence of Th17 lymphocytes in the milieu contributes to the persistence of inflammation. IL-6, TGF-β, and IL-1β are necessary cytokines for Th17 lymphocytes development, these cells secrete IL-17, IL-21, and IL-22. Combination of IL-17 with other cytokines such as IL-6 and IL-8 contributes to the chronicity of inflammation (54, 55).

An example of pathological angiogenesis during chronic inflammation is diabetic retinopathy (56). Angiogenesis in the retina of patients with diabetes is initiated by ischemia produced by chronic inflammation. In addition, the hyperglycemic environment activates a series of events, culminating in increased vascular permeability, the accumulation of extravascular fluid, ischemia, and pathological angiogenesis (57). Some studies have shown high levels of pro-inflammatory cytokines, including VEGF, TNF-α, NO, and IL-6 in the vitreous humor of patients with diabetes mellitus (57).

Another example is prolonged peritoneal dialysis. In this pathology, adipocytes secrete pro-inflammatory cytokines, which culminates in pathological angiogenesis. The association of chronic inflammation and angiogenesis also occurs in inflammatory bowel disease where continuous ulceration and regeneration lead to the development of chronic inflammation and pathological angiogenesis (58).

Further investigation of the association between inflammation and angiogenesis, which can result in a number of pathological conditions, is required for a better understanding of the underlying molecular events in these process. In the future, selected molecules may be useful as therapeutic targets for the reprogramming of homeostasis.

## Angiogenesis and Inflammation in Carcinogenesis

As discussed in the previous sections, increased vascular permeability during the inflammatory process is essential for the arrival of immune cells. The vast array of cytokines and chemokines that participate in the inflammatory process serve to activate and recruit immune cells, which also impacts the associated endothelial cells (59, 60).

Currently, the association of inflammation, angiogenesis, and cancer is well-known. Worldwide, 16% of cancers are caused by infections. In addition, 25% of all inflammatory processes are estimated to lead to tumor development (61). Unlike acute inflammation, during chronic processes of inflammation, inflammatory infiltrates consisting primarily of mononuclear cells that produce reactive oxygen and nitrogen species (RONS) are present. Most RONS have unpaired electrons; thus, they are considered free radicals. As such, while RONS are potent microbial agents, they can also cause cell damage when they are released. DNA is particularly sensitive to RONS, which can induce modified DNA structures such as 2'-deoxyribose. In particular, the modified DNA structure 7,8-hydroxy-2'-deoxyguanosine (8-oxodG) induces a breakdown in the double and single strand of DNA (62). These alterations can affect cell cycle regulation and lead to an increase in mutation rate. In driver genes, the gain of mutations has been shown to trigger carcinogenesis (63). Although this event in itself is not enough to produce a tumor, the resulting microenvironment favors chronic inflammation which is another factor involved in cancer initiation. Pro-inflammatory cytokines, such as IL-1 and IL-6, and growth factors, such as TGF-β and VEGF, then can activate various signaling pathways, primarily those involving NF-κB and STAT3 (64). It has been demonstrated that both NF-κB and STAT3 stimulate various survival signals in cells, associated with triggering the carcinogenesis process (53, 65). Other soluble factors generated in the inflammatory process include VEGF-A, cyclooxygenase-2, and prostaglandins. The overall effect of all these molecules on the endothelium is crucial for the recruitment of cells, the production of inflammatory mediators, the increase in vascular permeability, and angiogenesis (66, 67).

In 1986, Dvorak made the analogy of the tumor and its associated tumor microenvironment (TME) to a wound that does not heal (68). In this study, the tumor vasculature captured radiolabeled fibrinogen several-fold faster than did control tissue, allowing for an increase in tumor microvascular permeability. This increase in vascular permeability was attributed to the vascular permeability factor, now known as VEGF-A (68). In addition, Dvorak demonstrated that in the phenomena studied, ECM molecules, including laminin, fibronectin, collagen, and proteoglycans, were involved.

Another similarity between tumors and wound healing is the presence of inflammatory infiltrates. During these processes, cells release a plethora of angiogenic factors, including fibroblast growth factor (FGF)2, CXCL8,WNT7b, ANGPT2, IL-1β, IL-6, IFN-γ, CXCL9/10, and MMP2/9 (32).

In 2005, Cao et al. (69) transfected HCT116 colon carcinoma cells and T4 breast cancer cells with a hypoxia-responsive promoter. The cells were then inoculated in a rodent model using dorsal skinfold window chambers; the presence of angiogenesis was demonstrated at a very early stage of tumor development and was hypoxia-independent.

Mizukami et al. reported that inoculation of HIF-1α knocked down by siRNA in colon carcinoma cell lines reduced tumor growth with no alteration of angiogenesis in a CD1 nude mouse model (70). In addition, this same group demonstrated that under HIF-1α-independent angiogenesis conditions, the RAS and NF-κB signaling pathways upregulated the production of VEGF, IL-8, COX-2, and prostaglandin E (70).

Thus, the process of angiogenesis may be occurring at a very early stage of tumor development and not necessarily at the point of the hypoxia-induced angiogenic switch. However, a deeper research in this issue is necessary to design therapeutic schemes in order to impact in cancer patient clinical outcome.

## Angiogenesis and Inflammation in Cancer Establishment

It is widely known that hypoxia is another critical player in the tumor angiogenesis process. Several factors during cancer development contribute to the generation of hypoxia and the resulting VEGF release. The main molecular component of hypoxia-induced angiogenesis initiation is the hypoxia-inducible factor (HIF)-1α. In metazoan organisms, HIF-1α has been shown to play an essential role in oxygen homeostasis. Under normoxic conditions, HIF-1α is continuously synthesized and degraded. However, under hypoxic conditions, HIF-1α is stabilized and accumulates in the cytoplasm where it dimerizes with HIF-1β. The HIF-1α/HIF-1β complexes then control the expression of hundreds of genes, including VEGF (71). Hypoxia, in conjunction with angiogenesis, can also activate other cancer-specific biological pathways. Under hypoxic conditions, tumor cells present a metabolic shift from oxidative phosphorylation to aerobic glycolysis (72). In addition, hypoxia increases cellular proliferation and the avoidance of apoptosis, which contributes to the chemoresistance of tumors (71). Hypoxia also induces the fibroblasts surrounding the tumor, to acquire a cancer-associated fibroblast phenotype, which is associated with the release of bFGF, IL-6, PDGF, and TGF-β and favors a microenvironment conducive to the cellular evasion of the antitumor immune response (73). Hypoxia also induces the epithelial to mesenchymal transition (EMT), which encourages tumor cells motility, and MMP secretion, subsequently leading to an invasion phenotype (74). The angiogenic switch provides more advantages to the tumor than just angiogenesis, leading to the gradual acquisition of several tumor hallmarks, which allow the tumor to develop into more advanced stages (clinically advanced tumor).

## Angiogenesis and Inflammation During Cancer Metastasis

Metastasis is the leading cause of death from tumors. It can be described as the process by which tumor cells separate from the primary tumor, travel via the blood or lymph, and arrive at a distant site where they can establish a secondary tumor or metastasis.

In terms of a spatial-temporal context, after the angiogenic switch onset, the tumor establishes and grows. The resulting high rate of proliferation and mutagenesis then induces genetic heterogeneity in the tumor. Welch and Hurst proposed four characteristics of metastasis, namely, (i) Motility and invasion, (ii) microenvironment modulation, (iii) plasticity, and (iv) colonization. In addition to providing nutrients and oxygen, tumor angiogenesis also contributes to the metastatic cascade, which involves vasculogenic mimicry and co-option mechanisms (75).

Vasculogenic mimicry is the generation of structures such as channels and tubes, in conjunction with perfusion, and does not involve endothelial cells. The network formed by vasculogenic mimicry connects with blood vessels in order to supply blood and fluids to the tumor mass (76). Tumors that display vasculogenic mimicry are associated with greater aggressiveness and patients with these tumors typically have lower survival rates. In addition, vasculogenic mimicry is considered an evasion mechanism for antiangiogenic therapy (77, 78).

Within the motility/invasion phase of the metastatic cascade, a critical mechanism that allows tumor cells to acquire the necessary skills is the EMT. During embryogenesis, this mechanism is preponderant. However, in cancer progression, the EMT allows tumor cells to develop vasculogenic mimicry. As part of the EMT, VE-cadherin is expressed in tumor cells favoring both vasculogenic mimicry and metastasis. Moreover, inflammation associated with cancer contributes to both vasculogenic mimicry and the EMT (75, 78). Among the primary immune cells contributing to these mechanisms are tumor-associated macrophages (TAMs), which secrete MMPs for the remodeling of the ECM, favoring motility and tumor cells invasion. Furthermore, TAMs release an array of cytokines, including TGF-β, TNF-α, IL-1β, IL-6, and IL-8, which contribute to the activation of the EMT program (79, 80). Additional immune cells involved in these events include tumor-associated neutrophils (TANs) and myeloid-derived suppressor cells (MDSCs). This set of immune cells and the molecules they secrete then activate the PI3K and NF-κB signaling pathways for promoting the EMT and vasculogenic mimicry (81, 82). This perpetual tumor-associated inflammation and the ongoing redundancy of the factors released that gradually modulate the microenvironment undoubtedly impact the process of tumor progression.

A further important aspect is that tumor cells “appropriate” the pre-established vasculature during vessel co-option. This activity is not exclusive of angiogenesis but rather is considered as one mechanism of tumor cell invasion. Vessel co-option has been clinically associated with aggressive tumors, such as melanoma, glioblastoma, non-small cell lung carcinoma, and ovarian cancer (31, 83).

As observed throughout this review, the inflammatory response proceeds, or is intimately involved in the increase in vascular permeability and angiogenesis observed in both physiological and pathological processes. Indeed, the underlying inflammation in the tumor microenvironment promotes angiogenesis. Advantages conferred to the tumor by angiogenesis include an increase in cellular proliferation, metabolic reprogramming, invasion, and metastasis. Tumor angiogenesis also promotes the continuous arrival of immune cells at the site of the tumor. However, the changes in the tumor microenvironment at this step induce the immune cells to develop a phenotype that, instead of activating the antitumor immune response, favors tumor aggressiveness. As part of the tumor microenvironment, endothelial and immune cells, as well as tumor cells, continuously secrete VEGF (84). This growth factor has an immunosuppressive effect on some immune cells. Indeed, VEGF inhibits the maturation of dendritic cells (DC). In addition, it promotes the accumulation of MDSCs through the recruitment of monocytes/macrophages and, in addition with the IL-4 and IL-10 produced by tumor cells, induces the polarization to M2 macrophages (38).

In patients with colorectal cancer and advanced melanoma, a direct correlation between high concentrations of VEGF and Treg cells has been observed (85, 86). In a mouse model and in patients with colorectal cancer, a subpopulation of Treg cells expressing VEGFR-2 that expands with the exposition of VEGF has been reported (87). The tortuous blood vessels that the tumor develops as an outcome of angiogenesis, vasculogenic mimicry, and co-option all serve to prevent cytotoxic T-lymphocytes from reaching the tumor bed and exerting their antitumor action (88). In this case, the immune response acts promoting tumor growth.

## Antiangiogenic/Immunotherapy Combination

It has been reported that antiangiogenic therapy induces the “normalization” of the tortuous blood vessels that occur in pathogenic angiogenesis. Indeed, the combination of chemotherapy and antiangiogenic therapy appears promising and leads to increased survival of patients with cancer. This phenomenon is attributed to the normalization of blood vessels, which allows the chemotherapy drugs to reach the tumor bed (89). In addition, it has been demonstrated that radiotherapy in combination with antiangiogenic therapy leads to blood vessel normalization (90).

The first drug approved by the FDA for the antiangiogenic treatment of solid tumors was Bevacizumab, which is a humanized anti-VEGF monoclonal antibody. Bevacizumab, in combination with chemotherapy, has been shown to increase progression-free survival (PFS) and overall survival (OS) (91–95). Aflibercept is a recombinant protein known as a “VEGF trap” that can bind all VEGF isoforms, along with PLGF, and inhibit their activities. Patients with metastatic colorectal cancer have been treated with Aflibercept, with resulting increases in PFS and OS. Ramucirumab is a monoclonal antibody against VEGFR-2 that has been tested as a second line of treatment in combination with other chemotherapeutic agents. Sorafenib and Sunitinib are tyrosine kinase inhibitors that block VEGFR-2. In particular, Sorafenib is an inhibitor of multiple kinases and shows antiproliferative, apoptotic, antiangiogenic and antifibrotic properties. Sorafenib has been approved for hepatocellular carcinoma treatment. Sunitinib is also a multi-wide inhibitor approved for neuroendocrine pancreatic tumors and metastatic renal carcinoma (91, 96).

With respect to drugs that stimulate the immune system, several inhibitors of the various immunological checkpoints have been approved. The expression of the programmed death-ligand 1 (PDL-1) has been reported in tumor cells, macrophages, DC, and MDSCs. These cells bind to the programmed cell death protein (PD-1) on T-lymphocytes and inhibit their effect or function.

The cytotoxic T-lymphocyte-associated protein 4 (CTLA-4) receptor is another immune checkpoint regulated by hypoxia. In an oxygen-free environment, CD8+ T-lymphocytes increase the expression of CTLA-4, which binds to CD80 and CD86 present in antigen-presenting cells, resulting in the inhibition of CD8+ T-lymphocyte activity. PD1/PDL-1 and CTLA-4 represent therapeutic targets where their inhibitory activity can be blocked with the use of antibodies against these molecules. These kinds of immunotherapy favors the increase in T-lymphocytes to the tumor site and promotes their antitumor activity (88, 97–100).

Sustained angiogenesis and cancer-related inflammation share signaling pathways and molecules. New treatment strategies and the development of new drug combinations that inhibit angiogenesis and stimulate the antitumor response will undoubtedly lead to improved cancer treatments and patient survival in the near future.

## Concluding Remarks

This review was aimed to establish the relationship between inflammation and endothelial activation, which leads to increased vascular permeability and the initiation of angiogenesis. The relationship between these processes was reviewed for both non-tumor and tumor conditions.

In non-tumor conditions, soluble factors secreted by stromal and immune cells impact the endothelium and initiate its activation favoring the transmigration of cells to eliminate the harmful agent. The regulatory mechanisms of the oxidized phospholipids that contribute to the endothelium basal permeability state after acute inflammation were indicated. In addition, pathological angiogenesis during chronic inflammation were discussed.

The relationship between inflammation and angiogenesis in the advanced stages of cancer is supported by numerous studies. However, the few reports describing the association of these processes in the early stages of cancer are mentioned. It has been proposed that immune cells interact along with tumor development. Moreover, it has been suggested in the cancer immunoediting theory proposed by Dunn and Schreiber RD (101), that immune cells may interact with transformed cells for their elimination. When the eradication of the transformed cells does not occur, these cells gradually proliferate, increasing DNA mutations and the number of tumor cells. In this initial stage, more cells of the immune response arrive to the *in situ* tumor to eliminate only the susceptible tumor cells through their cytotoxic mechanisms. This premise was presented as the equilibrium phase of the immunoediting theory.

**Figure 1:**
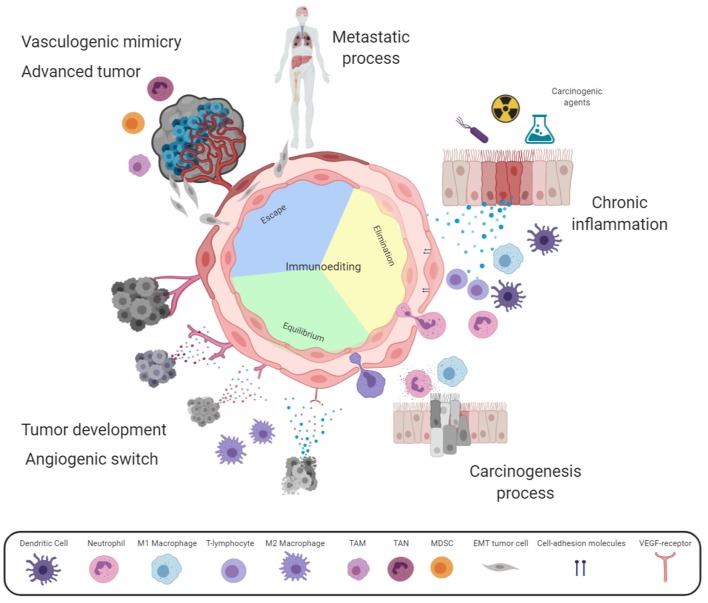
Angiogenesis involvement in chronic inflammation and cancer. Some harmful agents induce stress in resident cells releasing danger-associated molecular patterns (DAMPs), activating endothelial cells. Activated endothelium express adhesion molecules enabling immune cells extravasation for harmful agent elimination, and lastly tissue reparation. Whether the harmful agent is maintained, a positive feedback loop persists mediated by cytokines secreted by immune and stromal cells, causing chronic inflammation. In this case, more healthy tissue cells are damaged by the harmful agent or by reactive oxygen and nitrogen species (RONS) released by the emerging influx of leukocytes through vascular hyperpermeability. Sustained cellular damage may lead to carcinogenesis initiation. According to cancer immunoediting theory, immune cells recruitment might eliminate transformed cells (Elimination phase). However, in this complex microenvironment, some cytokines act as growth factors for transformed cells or in the endothelium increase vascular hyperpermeability and leukocyte transmigration. These immune cells destroy susceptible tumor clones (Equilibrium phase). Tumor development induces metabolic alterations leading to the angiogenic switch; while, immune cell infiltration now promotes tumor growth (Escape phase). At advanced cancer stages, tumor mass viability is maintained by sustained angiogenesis and vasculogenic mimicry. This complex and dynamic environment promotes phenotypic changes into aggressive tumors, which take advantage of the tortuous vascular branches generating metastatic foci. It should be noted that inside the endothelium circle, the three phases of the immunoediting cancer theory are indicated. The intensity of the color represents the gradual activation of the endothelium. Created with Biorender.com.

According to our point of view and based on this proposition, the angiogenesis process is required from the early development of an *in situ* tumor in order to favor the arrival of immune cells. For this purpose, the blood vessels adjacent to the incipient tumor increase their permeability to allow the transmigration of inflammatory cells to the tumor site. Therefore, it can be considered that tumor cell proliferation causes stress on the tumor-cell surroundings and the release of DAMPs. These molecules are then captured by receptors in both immune and endothelial cells which allows the endothelium activation and the arrival of inflammatory cells. The close relationship of these processes results in: (i) tumor cell proliferation, (ii) the release of DAMPs and pro-inflammatory cytokines, and (iii) endothelium activation and the recruitment of more inflammatory cells. This cyclic process gradually increases the region affected; thus, angiogenesis may contribute to the generation of a microenvironment that favors the presence of growth factors, secreted initially by the infiltrated cells and, tumor cell multiplication and genetic instability (see Figure 1).

Finally, owing to the high proliferation rate of tumors, hypoxia is induced; and the angiogenesis switch is turned on. The maintenance of this cyclic process further leads to cancer cells and the development of resistance mechanisms and evasion of the immune response. In this stage, the generation of a tumor microenvironment known as tumor-associated inflammation is induced. After this step, the established tumor can initiate the metastatic process, in which vasculogenic mimicry and co-option contribute to mechanisms of invasion and the migration of tumor cells. Tumor angiogenesis results in abnormal vasculature, with unstable, tortuous blood vessels uncovered by pericytes, which alter immune cell infiltration. Many patients with cancer are diagnosed at this advanced stage but due to the level of pathogenic angiogenesis, only the combination of antiangiogenic therapy and chemo/radiotherapy has been shown to increase the OS. A recent therapeutic option includes the combination of antiangiogenic therapy with inhibitors of various immunological checkpoints. This combination appears to “normalize” the abnormal blood vessels and favors the ability of T-lymphocytes to reach the tumor site and exert their antitumor activity (88).

In summary, sustained angiogenesis and cancer-related inflammation share important signaling pathways and molecules. These hallmarks ultimately serve to support tumor development. Therefore, improving the combination of therapies that inhibit pathological angiogenesis and stimulate the antitumor response may prove to be a successful strategy for the treatment of patients with cancer.

## Author Contributions

DA-C, RC-D, and JL-G organized the entire manuscript, wrote the draft, and revised the last version of the manuscript. AC-R, OH, and DA-C wrote the acute inflammation/vascular hyperpermeability. DA-C, CL-C, RC-D, and JL-G wrote the angiogenesis in chronic inflammation. AC-R, OH, and RC-D wrote the angiogenesis in the carcinogenesis process. Figure 1 was designed and made by DA-C, RC-D, and JL-G.

### Conflict of Interest

The authors declare that the research was conducted in the absence of any commercial or financial relationships that could be construed as a potential conflict of interest.
